# A systematic review of shared decision making interventions in child and youth mental health: synthesising the use of theory, intervention functions, and behaviour change techniques

**DOI:** 10.1007/s00787-021-01782-x

**Published:** 2021-04-22

**Authors:** Daniel Hayes, Julian Edbrooke-Childs, Rosa Town, Miranda Wolpert, Nick Midgley

**Affiliations:** 1 Evidence Based Practice Unit, University College London and Anna Freud National Centre for Children and Families, 4-8 Rodney Street, London, N1 9JH England; 2 Child Attachment and Psychological Therapies Research Unit (ChAPTRe), University College London and Anna Freud National Centre for Children and Families, 4-8 Rodney Street, London, N1 9JH England; 3 Health Services and Population Research Department, King’s College London Institute of Psychiatry, Psychology and Neuroscience, De Crespigny Park, London, SE5 8AF England

**Keywords:** Shared decision making, Child mental health, Youth mental health, Behaviour change, Interventions

## Abstract

Reviews around interventions to improve shared decision making (SDM) for child and youth mental health have produced inconclusive findings on what approaches increase participation. Importantly, the previous reviews did not explore the use of theory, as well as mechanisms of change (intervention functions) and active units of change (behaviour change techniques). The aim of this review was to explore these factors and ascertain how, if at all, these contribute to SDM. Five databases were searched up until April 2020. Studies met inclusion criteria if they were: (a) an intervention to facilitate SDM; (b) aimed at children, adolescence, or young people aged up to 25, with a mental health difficulty, or their parents/guardians; and (c) included a control group. Data were extracted on patient characteristics, study design, intervention, theoretical background, intervention functions, behaviour change techniques, and SDM. Quality assessment of the studies was undertaken using the Effective Public Health Practice Project (EPHPP) quality assessment tool. Eight different interventions met inclusion criteria. The role of theory to increase SDM remains unclear. Specific intervention functions, such as ‘education’ on SDM and treatment options and ‘environmental restructuring’ using decision aids, are being used in SDM interventions, as well as ‘training’ for clinicians. Similarly, behaviour change techniques linked to these, such as ‘adding objects to the environment’, ‘discussing pros/cons’, and clinicians engaging in ‘behavioural practice/rehearsal’. However, as most studies scored low on the quality assessment criteria, as well as a small number of studies included and a low number of behaviour change techniques utilised, links between behaviour change techniques, intervention functions and increased participation remain tentative. Intervention developers and clinicians may wish to consider specific intervention functions and behaviour change techniques to facilitate SDM.

**Supplementary Information:**

The online version contains supplementary material available at 10.1007/s00787-021-01782-x.

## Introduction

The last 50 years have seen a shift from the paternalistic model of health, towards one, where patients are actively involved in shaping and developing healthcare [1]. This can occur across different levels of the healthcare system including service redesign, where patients play a role in reviewing and developing interventions, through to treatment decision making [1]. This concept, referred to a shared decision making (SDM), acknowledges that both clinicians and patients have expertise which is important [2]. In the context of making decisions around an individuals’ own care and treatment, the clinician brings their professional knowledge and clinical experience, whilst the patient brings a lived experience of their illness and what would fit with their lifestyle [3]. Through joint communication, understanding and deliberation, both parties should arrive at an option for care and treatment which they deem acceptable [4].

More recently, the concept of SDM has been applied to children and young people [5]. Involvement in such decisions is enshrined in the United Nations Convention on the Rights of the Child [6]. Here, articles 12 and 13 are particularly relevant to care and treatment decisions, outlining that the views and opinions of the child should be given consideration in line with their age and maturity. In treatment decisions, whilst this may not mean that the child or young person has ultimate decision-making power, it does highlight that at a minimum, they should be allowed to express their own views and opinions and have these taken into consideration [7].

Models have been developed to try and better facilitate SDM in clinical practice [4, 8–11]. These conceptualise different aspects of SDM as skills, competencies and behaviours that can be taught to those involved in the decision-making process. One of the most widely cited models, an integrative model of SDM, identified 13 elements which should be present, as well as 10 general qualities which clinicians should have [10]. However, this model was developed from literature mainly situated in adult physical health, meaning that key aspects may have been missed when involving children and young people with mental health difficulties.

In the field of child and youth mental health, both generic [12, 13] and context specific [14, 15], models have been developed. Many have core overlapping features, such as discussing values, preferences, and options, as well as arranging follow up [16]. However, differences also exist, such as whether they are aimed at parents/guardians or young people, and whether there needs to be explicit agreement among all stakeholders prior to a decision being made [16].

In addition to models, a number of interventions to facilitate SDM in child and youth mental health have been developed. These have been categorised into six overarching approaches: therapeutic techniques, psychoeducational information, decision aids, action planning or goal setting, discussion prompts, and mobilising patients to engage [17]. Reviews exploring the effectiveness of approaches have produced inconsistent results, with some interventions being effective in improving participation in decision making in certain circumstances [17, 18].

Two important limitations exist which may account for these differences in effectiveness of different approaches [16] First, whether the interventions used theory was not examined. The use of theory is important as it not only allows for the identification of causal determinants of change and mediators, but also allows a space in which theories can be tested and evaluated [19]. Reviews of interventions across healthcare settings indicate that the use of theory can lead to more effective outcomes [20, 21]. Within the field of SDM, there is tentative support for this notion, where computerised decision aids underpinned by theory were more likely to lead to increases in participation [22].

Second, grouping interventions by overarching approach neglects the unique features within each, which may cause individuals to behave in different ways. If, as models and experts suggest, SDM is a set of behaviours or skills that can be taught to stakeholders [10, 23–26], then it is important, within each approach, to understand the specific ways in they attempt to change behaviours to facilitate SDM.

The behaviour change wheel is an amalgamation of 132 different behaviour change constructs and is an ideal lens in which to explore SDM behaviour [16]. Within this, interventions may be broken down into both intervention functions and behaviour change techniques [27]. Identification functions refer to the underlying causal mechanisms of change responsible for changing behaviour. Nine different intervention functions exist: ‘Education’, ‘Persuasion’ ‘Incentivisation’, ‘Coercion’, ‘Enablement’, ‘Training’, ‘Modelling’, ‘Environmental Restructuring’, and ‘Restriction’. In the context of SDM, ‘Education’ could refer to increasing patient knowledge around options, whilst ‘Training’ could be where clinicians are taught how to elicit preferences.

Further to the nine intervention functions, 93 behaviour change techniques also exist. These refer to the smallest components of behaviour change interventions that, on their own and in favourable circumstances, can bring about change [27]. For SDM, examples of these could be incorporating the use of a decision aid into the clinical encounter; which would correspond to the behaviour change technique ‘adding objects to the environment’. Whilst the comparison of different options on the decision aid would map onto the behaviour change technique ‘pros/cons’.

A recent study drawing on secondary data analysis from a 2014 Cochrane review [28] has explored the role of behaviour change techniques in SDM [29]. In the 87 included interventions, 7 different intervention functions and 32 behaviour change techniques were identified. Within this, the most common intervention function used was ‘education’ and the most common behaviour change technique was ‘information about health consequences. Whilst this is useful in providing an initial framework, there were no included interventions in child and youth mental health. Given the unique properties of this population, such as multiple stakeholders and capacity due to age and having a mental health difficulty[30], establishing behaviour change techniques within this population is needed.

Inspecting intervention functions and behaviour change techniques may allow researchers and intervention developers to better understand the drivers of change that are present in tools that facilitate SDM for care and treatment decisions. Given the above, this study will undertake a review of the literature and explore the impact of theory, intervention functions, and behaviour change techniques on SDM around patient treatment decisions in child and youth mental health.

Specific research questions:

1. What theory is being used to facilitate SDM in child and youth mental health?
2. What intervention functions are being used to facilitate SDM in child and youth mental health?
3. What behaviour change techniques are being used to facilitate SDM in child and youth mental health?
4. Does the inclusion of the above aspects lead to increased SDM in child and youth mental health?

## Method

A team of individuals with a knowledge of SDM in child and youth mental health was convened. Expertise and knowledge included winning bids and writing papers on SDM in child and youth mental health (DH, JEC, MW), developing models of SDM in child and youth mental health (DH, MW), advising on child and youth mental health service transformation, where SDM is a central component (DH, JEC, MW), delivering training to clinicians on SDM practice (DH, RT, MW), and developing decision aids and tools to facilitate SDM in child and youth menta health (DH, JEC, RT, MW, NM).

Five research databases were searched up until April 2020—PsycINFO, EMBASE, Medline/PubMed, Web of Science and Cochrane Libraries. The search strategy included three concepts: ‘SDM’, ‘child, adolescent, or young person (up to the age of 25, or their parent/guardian), and ‘mental health’ (including both diagnosable and non-diagnosable menta health difficulties). The search strategy is included in the supplementary material. Eligibility criteria are outlined in Table 1 and were developed in line with the research questions. Studies were limited to English language and peer reviewed publications. Database searching was not limited to a particular timeframe.

**Table 1:** Inclusion/exclusion criteria

	Inclusion criteria	Exclusion criteria
Population	A child or young person (up to the age of 25) with a diagnosable or non-diagnosed mental health difficulty, or their parent/guardian	Studies where the presenting difficulty is physical health
Intervention	Any intervention, approach or tool (e.g., online decision aids, mobile applications and training) aimed at facilitating decision making around care and treatment	Interventions whose primary aim is not facilitate in care and treatment decisions
Comparator	Studies where an there is an intervention and control arm. This can include non-randomised control studies	Studies where there is no control arm
Outcome	Includes a measure examining the process of SDM (e.g., using the SDM-Q-9 (Kriston et al., 2010) or CollaboRATE (Elwyn et al., 2013). Unvalidated measures will be included. Outcomes can be reported by any individual (e.g., child/ young person, parent/guardian, healthcare professional)	Includes only an outcome measure related to SDM (e.g., decisional conflict) without also including a process measure. This is because measuring decision outcome is not a meaningful indicator of quality, as the eventual outcome can be dependent upon many external factors (Elwyn, Elwyn, & Miron-Shatz, 2009)
Study Design	Randomised and clinical control studies	Qualitative studies and case studies. Studies not reported in English. Conference presentations will be excluded as these have been found to differ substantially from peer-reviewed papers on outcome metrics (Balshem et al., 2013)
Other	English language Any date/timeframe	Language other than English N/A

**Fig. 1:**
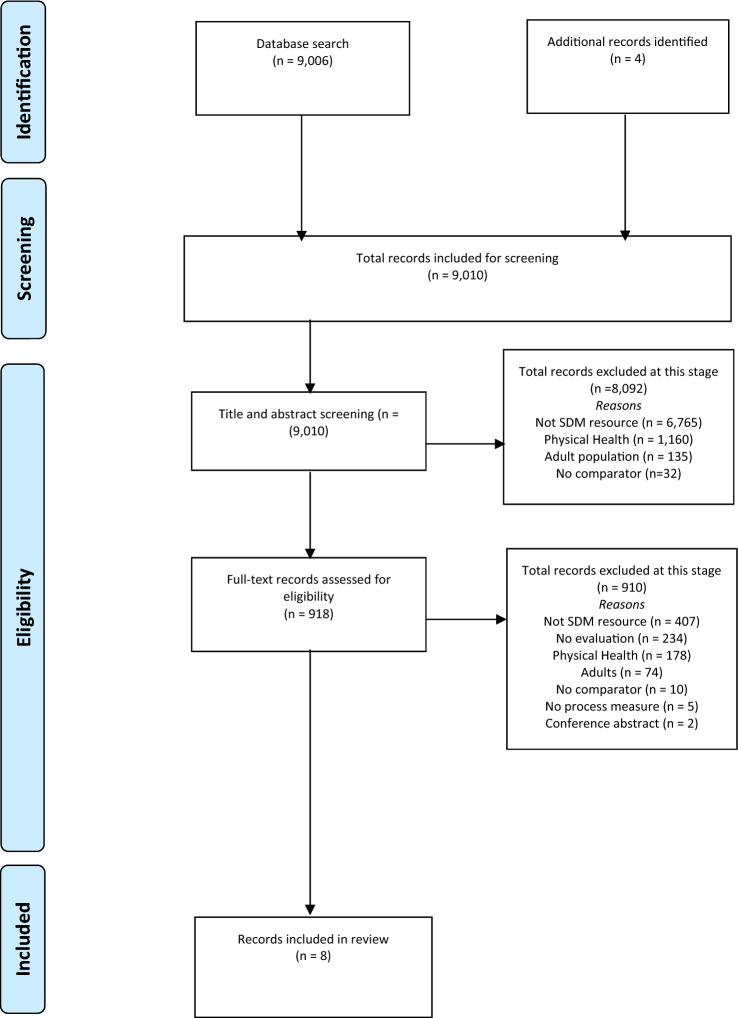
PRIMSA flowchart

To identify additional records, reference checking of the following articles was undertaken: (a) those at second stage screening that focused on SDM and children and young people but had no evaluation (*n* = 234) and (b) those that met full inclusion criteria. In addition, consultation with researchers in the field of SDM via an online Facebook group and at the International Shared Decision-Making (ISDM) conference during a child and youth mental health panel. The study selection was completed using a two-stage process by two researchers (DH, RT). The first stage involved screening article titles and abstracts, during which all records were screened by the first author (DH) and 10% by the second author (RT) and any results that were not relevant were excluded. The second stage consisted of full-text screening by both authors. A good inter-rater reliability was found at both first- and second-stage screening (0.78 and 0.87), respectively. The exclusion of papers at each stage is highlighted in Fig. 1. For each included article, data were extracted independently by the same two researchers reading articles and available documentation line by line and extracting data using a template. This included author, year and publication date, participant details, study design, intervention, theoretical background, intervention functions and who they were aimed at, behaviour change techniques and who they were aimed at, as well as any SDM process measures. For behaviour change techniques and intervention functions, both researchers involved in the data extraction process completed an online training (https://www.bct-taxonomy.com/).

Authors and intervention developers of resources and papers deemed acceptable for inclusion were contacted to establish whether any further information on the intervention component was available (e.g., a manual or protocol). For extracted intervention functions and behaviour change techniques, a good level of agreement was obtained between the researchers extracting data (Kappa = 0.81 and 0.90, respectively). Any discrepancies were resolved by discussion and agreed upon by the researchers. The finalised intervention functions and behaviour change techniques were submitted to two research psychologists working in behaviour change. From this, one additional behaviour change technique, ‘credible source’, was included on some records.

Studies were quality assessed using the Effective Public Health Practice Project (EPHPP) Quality Assessment Method [31] which is acceptable for examining both randomised and non-randomised studies [32]. This explores the risk of bias within studies on the following domains: selection bias, study design, confounding variables, blinding, data collection methods, and withdrawal and drop out. Each section is given a rating: strong, moderate or weak, and from this an overall rating is calculated. Each study that met inclusion criteria was quality assessed independently by two researchers (DH & RT). A good level of agreement was obtained between the researchers (Kappa = 0.82). Any discrepancies were resolved by discussion and changes were agreed upon by both researchers.

## Results

Database and hand searching returned 9010 articles. The screening of titles and abstracts (first stage screening) resulted in the exclusion of 8092 records. Next, full-text screening (second stage screening) resulted in the exclusion of 910 results. A total of eight studies met the inclusion criteria for this review. Their characteristics, including behaviour change techniques, intervention functions, process, and outcome measures, are shown in Table 2.

**Table 2:** Characteristics of interventions included in the final review

Included article number	Author, year of publication, and country	N	Comparison and design	Intervention and theoretical background	Intervention function(s)	Behaviour change techniques	Process measure: decision making/involvement/ participation	Results
1	Aoki et al., (2020), Japan	88 young people with a mood (depression or bipolar) disorder	Intervention vs usual practice Randomised control trial	Three decision aid booklets (depression, bipolar disorder, and medication treatment) No theoretical background specified Followed IPDAS^1^	With young people ‘Enablement’ With healthcare workers ‘Training’ With both ‘Education’ ‘Environmental restructuring’	With young people 5.1 ‘Information about health consequences’ 9.1 ‘Credible source’ 9.2 ‘Pros/cons’ For healthcare workers 4.1 ‘Instruction on how to perform behaviour’ 8.1 ‘Behavioural practice/rehearsal’ 8.3 ‘Habit formation’ Both young people and healthcare workers 12.5 ‘Adding objects to the environment’	^^^SDM (COMRADE; Edwards et al., 2003: YP rated)	^Ø^Duration of consultation (Researcher rated) ^Ø^Satisfaction (YP rated) ^Ø^Looked up treatment after (YP rated) ^Ø^Discussed options with others outside clinic (YP rated) ^Ø^Depressive symptoms at 3 and 6 months (YP rated) ^Ø^Persistence of treatment (audit records) ^Ø^Medication adherence (YP rated)
2	Brinkman (2013), US	44 parents/guardians of young people with ADHD, 7 paediatricians	Intervention vs usual practice Controlled clinical trial	Pre-encounter cards, booklet, DA, and healthcare worker training No theoretical background specified Followed IPDAS^1^	With healthcare workers Training’ ‘Modelling’ Both parents/guardians and healthcare workers ‘Environmental restructuring’ ‘Enablement’ ‘Education’	With healthcare workers 6.1 ‘Demonstration of behaviour’ 7.1 ‘Prompts/cues’ With parents/guardians 1.3 ‘Goal setting (outcome)’ 4.1 ‘Instruction on how to perform behaviour’ 5.1 ‘Information about health consequences’ 9.1 ‘Credible source’ 9.2 ‘Pros/cons’ Both parents/guardians and healthcare workers 12.5 ‘Adding objects to environment’	˄ SDM (Option Scale; Elwyn et al., 2005: O rated)	^˄^Knowledge (P/G rated) ^Ø^Decisional conflict* (P/G rated) ^Ø^Follow up calls and visits (audit records) ^Ø^Prescriptions written (audit records) ^Ø^Behavioural ratings (P/G and T rated) ^Ø^Titration of medication audit records) ^Ø^Number of days covered (audit records) with medication ^Ø^Physician satisfaction with choice (C rated)
3	Grant (2016), Australia	81 parents/guardians of young people with autism	Intervention vs usual practice	An online decision aid outlining treatments for Autism No theoretical background specified Followed IPDAS^1^	With parents/guardians ‘Education’ ‘Enablement’ ‘Environmental restructuring’	With parents/guardians 9.2 ‘Pros/cons’ 12.5 Adding objects to the environment	^Ø^Decisional conflict^#^ (Support subscale: P/G rated)	^Ø^Parental Sense of Competency Scale (PSOC) [42] (P/G rated)
4	Hogue et al. (2016), US	3 MIP therapists and 35 young people with ADHD and their parents/guardians	Intervention vs historical control Controlled clinical trial	A therapeutic approach (MIP) promoting family decisions about medication No theoretical background specified	With young people and parents/guardians ‘Education’ ‘Enablement’ With healthcare workers ‘Education’ ‘Training’	With young people and parents/guardians 1.2 ‘Problem solving’ 1.3 ‘Goal setting (outcome)’ 1.5 ‘Review behavioural goals’ 1.7 ‘Review outcome goals’ 5.1 ‘Information about health consequences’ 5.3 ‘Information about social/environmental consequences’ 9.1 ‘Credible source’ 9.2 ‘Pros/cons’ 13.2 ‘Framing/ reframing’ With healthcare workers 4.1 ‘Instruction on how to perform behaviour’ 8.1 ‘Behavioural practice/rehearsal’ 8.3 ‘Habit formation’	^^^Family decision making (O rated) Non validated measure	^^^Psychiatric evaluation completion (audit records) ^^^Prescribed any medication (audit records) ^^^Prescribed ADHD (audit records) medication ^Ø^Days on ADHD medication (audit records)
5	Rowe et al., (2018) UK	23 young people with self harm	Intervention vs usual practice Randomised Control Trial	An online decision aid for self-harm support No theoretical background specified	With young people: ‘Education’ ‘Enablement’ ‘Environmental restructuring’	With young people 9.2 ‘Pros/cons’ 12 5 ‘Adding objects to the environment’	^Ø^Decisional conflict ^#^ (Support subscale: YP rated)	^Ø^Intended help seeking (YP rated) ^Ø^Actual help seeking (YP rated)
6	Simmons et al., (2017) Australia	149 young people ages 16–25 (presenting difficulty not specific),	Intervention vs historical control Controlled clinical trial	Peer support worker and decision support tool No theoretical background specified Followed ODSF and IPDAS	With young people and healthcare workers : ‘Education’ ‘Enablement’ ‘Environmental restructuring’ For healthcare workers ‘Training’ ‘	With young people 3.1 ‘Social support (unspecified) 9.1 ‘Credible source’ 9.2 ‘Pros/cons’ 12.5 ‘Adding objects to the environment’ For healthcare workers 4.1 ‘Instruction on how to perform behaviour’ 8.1 ‘Behavioural practice/rehearsal’ 8.3 ‘Habit formation’ 9.1 ‘Credible source’ 12.5 ‘Adding objects to the environment’	^^^SDM (SDM-Q-9-[43] (YP rated)	^Ø^Decisional conflict (YP rated) ^Ø^Satisfaction with service (YP rated)
7	Walker et al., (2017). US	55 high risk young people with mental health difficulties (not specific). Involved in at least two systems designed to support young people (mental health and child welfare)	Intervention vs usual practice Randomised Control Trial	A wraparound service for young people aimed at increasing collaboration and participation in care No theoretical background specified	With young people ‘Enablement’ With healthcare workers ‘Education’ ‘Training’	With young people 1.2 ‘Problem solving’ 1.3 ‘Goal setting (outcome)’ 1.7 ‘Review outcome goals’ 3.2 ‘Social support (practical)’ 9.1 ‘Credible source’ With healthcare workers 4.1 ‘Instruction on how to perform behaviour’ 8.1 ‘Behavioural practice/rehearsal’ 8.3 ‘Habit formation’	^^^Participation Youth Participation in Planning Scale (YPP; Walker and Powers 2007) (YP rated)	^^^Youth Participation in planning (YPP) Preparation (YP rated) ^^^YPP Planning ^Ø^YPP accountability (YP rated) ^^^Working Alliance Inventory (WAI; Horvath and Greenberg 1989) (YP rated)
8	Westermann et al., (2013). Netherlands	71 parents/guardians of young people (presenting difficulty not specific), and 20 therapists	SDM vs usual practice: Randomised Control Trials	Counselling in Dialogue No theoretical background specified Followed the ODSF^2^	With healthcare workers ‘Training’ ‘Modelling’ Both healthcare workers and parents/guardians ‘Environmental restructuring’ ‘Education Enablement’	With healthcare workers 8.1 ‘Behavioural practice/rehearsal’ 8.3 ‘Habit formation’ With parents/guardians 9.1 ‘Credible source’ Both parents/guardians and healthcare workers 1.2 ‘Problem solving’ 9.2 ‘Pros/cons’ 12.5 ‘Adding objects to the environment’	^^^Satisfaction with participation in shared decision making (mothers)	^Ø^Decisional conflict (C rated) ˄ Decision made with accurate information (P/G rated) ˄ Accepting recommended treatment (P/G rated) ^Ø^Consensus on diagnostic formulation (P/G and C rated)

^^^Measure increased, ^˅^Measure decreased, ^Ø^No change on measure, ^1^International Patient Decision Aid Standards (IPDAS)

^2^Ottawa Decision Support Framework, *Authors report this finding as significant at *p* < 0.06, ^$^Parents/guardians were allocated to choose a treatment of their choice, or be randomly allocated a treatment (no choice). ^#^The decisional conflict scale (O’Connor, 1995) is both a process and outcome measure for SDM [44]. *YP rated* young person rated, *P/G rated* parent/guardian rated, *O rated* observer rated, *T rated* teacher rated, *C rated* clinician rated

Of the eight interventions designed to increase SDM in child and youth mental health settings, four were aimed for young people as the decision maker [33–36], three for parents/guardians [37–39], and one for both parents/guardian and young people [40]. In terms of overarching approaches to facilitate SDM, five interventions included decision aids [33–35, 39, 41] and three were therapeutic approaches [36, 38, 40]. Three papers came from the United States (US) [36, 40, 41], two from Australia [34, 39], one from the United Kingdom (UK) [35], one from the Netherlands [38], and one from Japan [33]. In terms of presenting difficulties, three SDM approaches were not specific to a particular difficulty [34, 36, 38], two focused on Attention deficit hyperactivity disorder (ADHD) [40, 41], one focused on self-harm [35], one focused on depressive symptoms [33], and one focused on autism [39].

### The theory used in interventions to facilitate SDM in child and youth mental health

None of the interventions explicitly outlined using one specific theoretical framework. One intervention [38] followed the Ottawa Decision Support Framework (ODSF), which is a framework that incorporates multiple theories [42]. For the ODSF, this includes: expected utility theory [43], decision analysis [44], prospect theory [45], the conflict theory model of decision making [46], the theory of reasoned action [47], self-efficacy [48], and factors related to social support [49, 50]. Three interventions [33, 39, 41] specified that they had used the International Patient Decision Aids Standards (IPDAS) guidelines for developing interventions. The IPDAS guidelines draw on some theory to ensure that relevant content is included when developing decision aids [51], including expected utility theory [43] and prospect theory [45]. One intervention outlined the use of both the ODSF and IPDAS guidelines in intervention development [34].

### Behaviour change techniques used in SDM interventions in child and youth mental health

Overall, 18 behaviour change techniques were identified across the eight interventions. The number of different behaviour change techniques per intervention ranged from two to 11, with a median of 7 (IQR = 5–7.5). The most frequently used behaviour change technique was ‘pros/cons’ which appeared in seven interventions and refers to the weighing up of different options with the clinician or using a decision aid [33–35, 38–40, 52]. This was followed by ‘credible source’, which appeared across six interventions and refers to the clinician, peer worker, or coach, providing advice based on their expertise [33, 34, 36, 38, 40, 52]. Similarly, ‘adding objects to the environment’ appeared in six interventions. This included the use a decision aid in five instances and the use of a visualisation aid in the remaining intervention [33–35, 38, 39, 52].

The three ‘Behavioural practice/rehearsal’, ‘habit formation’, and ‘instructions on how to perform the behaviour’ each appeared in five interventions and refer to a clinician, peer worker, or coach learning about and practicing using the decision aid or the therapeutic approach [33, 34, 36, 40, 52]. ‘Information about health consequences’ appeared in three interventions and refers to the decision aid or clinician facilitating SDM by providing the risks or side effects of options [33, 40, 41]. ‘Problem solving’ appeared three times when there was explicit discussion between stakeholders in identifying patient difficulties [36, 38, 40], whilst both ‘goal setting’ and ‘reviewing outcome goals’ appeared twice [36, 40].

### Intervention functions used in SDM interventions in child and youth mental health

Across the eight interventions, five different intervention functions were identified. These included: ‘Education’, ‘training’, environmental restructuring’, ‘modelling’, and ‘enablement’. Per intervention, the number of intervention functions ranged from three to five, with a median of 3.00 (IQR = 3.5–4.25).

The most frequent intervention function was ‘education’, which was identified across all interventions and refers to patients receiving information about options and risks and clinicians learning about SDM and how to facilitate it during appointments. ‘Enablement’ was also identified across all eight interventions. This refers to focusing on setting goals and exploring clinician and patient beliefs.

‘Training’ was found in six interventions and refers to clinicians, peer workers and coaches learning SDM skills [33, 34, 36, 38, 40, 41]. ‘Environmental restructuring’ was also present in six interventions and refers to the use of decision aids or visual aids [33–35, 38, 39, 52]. ‘Modelling’ was found in two interventions and refers to clinicians being shown how to use tools or approaches and then attempting to replicate that behaviour [38, 41].

### Relationships between intervention functions, behaviour change techniques, and SDM in child and youth mental health

The next section explores the relationship between intervention functions, behaviour change techniques, and SDM. Supplementary Tables 1 and 2 indicate the intervention functions and behaviour change techniques present within each study and whether an increase in SDM was found.

The heterogeneity of process measures and populations precluded the pooling of results for meta‐analysis. Six interventions reported a statistically significant^1^ increase for participation in decision making [33, 34, 36, 38, 40, 41], whilst two did not [35, 39]. As only two different intervention approaches were identified, each approach will be explored to see if specific behaviour change techniques, intervention functions, and theory impact on participation in SDM.

### Behaviour change techniques and increased participation in decision making in child and youth mental health

#### Decision aids

For decision aids, the behaviour change techniques ‘adding objects to the environment’, ‘pros/cons’, and ‘credible source’ showed the most promise in facilitating SDM (indicated by a statistically significant increase in the process measure utilised). However, these techniques were only successful when used in conjunction with other behaviour change techniques [33, 34, 41]. These will be described below:

For young people or parents/guardians, these behaviour change techniques included: ‘information about health consequences’, ‘information about social/environment consequences’, and ‘goal setting’ [33, 34, 41]. Whilst for clinicians or peer workers using decision aids, these included: ‘instructions on how to perform the behaviour’, ‘behavioural practice/rehearsal’, and ‘habit formation’ appeared to enhance SDM when used in conjunction with ‘adding objects to the environment’, ‘pros/cons’, and ‘credible source’.

#### Therapeutic approaches

All therapeutic approaches improved participation in SDM [38, 40, 41]. Those that were used the most frequently between stakeholders, and provided the most evidence for increasing SDM (indicated by a statistically significant increase in the process measure utilised), included ‘problem solving’, ‘pros/cons’, and ‘credible source’. For clinicians and peer workers, ‘behavioural practice/rehearsal’ and ‘habit formation’ also showed promise.

### Intervention functions and increased participation in decision making in child and youth mental health

#### Decision aids

For decision aids, the intervention functions ‘education’, ‘environmental restructuring’, and ‘enablement’, aimed for young people, parents/guardians and clinicians/peer workers, were found to increase SDM when also paired with ‘training’ for clinicians and peer workers.

#### Therapeutic approaches

For therapeutic approaches, ‘education’ and ‘enablement’ used with all stakeholders involved in the decision-making process facilitated SDM. ‘Training’ for clinicians and the health coaches also had evidence for increasing SDM.

### Linking participation with wider outcomes

The wide range of outcome measures employed and differences in whether interventions increased participation in shared decision making makes drawing further conclusions difficult. One metric common across two studies was whether the young person was satisfied with treatment [33, 34]. In both these instances, significant increases in shared decision making were found; however, neither resulted in increased satisfaction. Similarly, another metric, again found in two studies, was prescriptions written [40, 41]. Similar to the previous example, whilst increased participation in decision making was found, this did not translate through a change in prescriptions written.

### Quality assessment for risk of bias

The results from the EPHPP quality assessment are depicted in Supplementary Table 3. Of the eight studies, one was rated strong overall, as indicated by no weak ratings across any of the EPHPP criteria. Two were rated as moderate overall, as indicated by one weak rating across all quality assessment criteria. Finally, five studies were rated as weak overall as they scoring two or more weak ratings in total. The categories ‘study design’ and ‘data collection methods’ received the highest frequency of strong ratings, whilst ‘controlling for confounding variables’ and ‘making sure outcome assessors were blinded’ received the highest frequency of weak ratings.

## Conclusions and discussion

The aim of this review was to explore the impact of theory, intervention functions, and behaviour change techniques on SDM in child and youth mental health.

### Use of theory in interventions

Two interventions, both which were therapeutic approaches, were not underpinned by theory. One therapeutic approach was underpinned by the ODSF, four decision aids utilised the IPDAS guidelines [51], and one decision aid used both the IPDAS and ODSF. Whilst both frameworks are described as being underpinned by theory, the degree to which the IPDAS is completely theoretically informed has been questioned by some [53, 54]. In particular, critics have stated that the documentation related to the IPDAS guidelines section, ‘presenting probabilities in an unbiased and understandable way’, is ill-defined, not conceptually clear, and lacking in both theoretical and empirical support [53]. These concerns have been echoed by others, with academics suggesting that the IPDAS guidelines should be considered critically from both theoretical and empirical perspectives [54].

If the IPDAS guidelines are included as a theory-led framework, the proportion of interventions reported here that incorporate theory is higher than in the previous research [22, 55, 56]. However, unlike the previous reviews, no individual theories were used to develop tools to facilitate SDM. This may highlight the growing recognition of the theory–practice gap, which states that relying on an individual theory will neglect other factors, such as cognition, the environment, or the tools themselves [57]. The ODSF is one solution to this, as it incorporates multiple theories. However, the Theoretical Domains Framework [58] should also be considered, as it incorporates a greater number of theories and may be used flexibly to change SDM behaviour through targeting capability, opportunity, and motivation [16].

### Behaviour change techniques

Overall, 18 out of 93 possible behaviour change techniques were identified in SDM interventions in child and youth mental health. This is less than the 32 found in the previous review [29]; however, as the previous review did not limit itself to any specific presenting difficulty nor population, a wider range of behaviour change techniques may be expected.

Of the behaviour change techniques found in this review, some explicitly map onto the integrative framework of SDM [10], such as ‘pros/cons’, whilst others overlap with constructs in the integrative framework, such as ‘credible source’, which corresponds with ‘professional knowledge’. In addition, behaviour change techniques, such as adding ‘objects to the environment ‘, whilst not appearing in such frameworks, lend themselves well to SDM, as young people and parents/guardians often report a lack of appropriate sources to help facilitate decision making [59, 60].

In the previous review exploring behaviour change techniques [29], the most frequently used behaviour change technique used was ‘information about health consequences’. In this review, the most common behaviour change technique was ‘pros/cons’. This difference could highlight the importance of other factors, beyond pure health outcomes when making a decision in child and youth mental health. For example, research suggests that other factors, such as financial, educational and social, are important to the patients and families [61], as well as their goals, values and preferences [10]. However, it is important to note that whilst identified behaviour change techniques differ in frequency, there appears to be considerable overlap between common behaviour change techniques used in both reviews (e.g., ‘demonstration of behaviour’ for healthcare professionals). This may reinforce the potential of a core taxonomy of behaviour change techniques common to SDM.

Of interest is the use of three behaviour change techniques: ‘adding objects to the environment’, ‘pros/cons’, and ‘credible source’ When these techniques were incorporated into decision aids and used by young people and parents/guardians outside the clinical appointment, they did not appear to increase involvement in SDM. This highlights the expertise of both patients/carers and those within clinical settings and the importance of arriving at a joint decision via discussion. Whilst factors, such as time, have been highlighted as an issue by clinicians when it comes to SDM [62, 63], providing tools for use outside of the clinical session may not be preferable if these tools are not subsequently discussed in the clinic.

### Intervention functions

Five intervention functions were identified as having the potential to increase SDM. The most frequently used intervention functions in this review were ‘education’ and ‘enablement’. This suggests that intervention developers may think individuals lack the knowledge and motivation required to participate engage in SDM and require support in overcoming these barriers.

In the previous review exploring intervention functions [29], education was the most common, followed by enablement, the discovery that ‘education’ was an intervention function linked to increased participation in decision making is supported by previous reviews of patient behaviour change interventions [64]. Moreover, there is support in the wider literature for educating and enabling individuals as a method of increasing participation in SDM. For example, a Cochrane review of the use of decision aids across healthcare settings found that these tools educated and enabled individuals in the following ways: they improved patients’ knowledge of treatment options, they helped patients understand what mattered most to them, they provided patients with more accurate expectations of the risks and benefits for options, and they helped patients to participate more in decision making [65].

The previous review also identified training, usually with clinicians, as a common intervention function [29]. The use of training to bring about behaviour change is also frequently reported in the literature when designing interventions [64]. Indeed, findings from clinicians in child and youth mental health services who have tried to incorporate new SDM tools and techniques into the doctor–patient encounter report feeling apprehension at the start of the process [66]. Clinicians also reported that prior to SDM tools being incorporated into their practice, there was a stage of ‘feeling clunky’ [66]. This could suggest that further training, as well as modelling, may be useful in expediting the acceptance of tools and techniques in clinical practice.

### Wider findings

It is too early to establish how participation in decision-making links with wider outcomes. This is due to a very small sample of overlapping outcomes, both of which tentatively suggest that increased participation in decision making had no effect on satisfaction or prescriptions written. This fits with the wider literature in adult mental health, where research on this topic is also inconclusive [67]. Whether or not shared decision making results in additional positive benefits, it is important to remember that many young people feel powerless and left out of care and treatment decisions, and that any intervention that facilitates this should be welcomed, as their right for involvement enshrined in the UN rights of the child [6].

Previous reviews have highlighted that the majority of approaches and evaluations to facilitate SDM focus on difficulties, such as ADHD and autism [17, 18]. Within this review, the range of difficulties targeted by interventions appears to be more diverse, as it includes self-harm and depressive symptoms. This is a welcome development as research suggests that lower levels of SDM may be related to the severity rather than type of difficulty [16, 68]. However, caution should be taken when translating interventions into UK settings, particularly as most of these interventions were developed in the US and Australia, which place a greater emphasis on insurance within healthcare. This is evident in resources, such as the decision choice cards, in which cost has its own card and prices are outlined for each treatment [41].

### Strengths and limitations

This is the first study to examine theory, intervention functions, and behaviour change techniques for SDM approaches in child and youth mental health. A strength of this study is that it examines the intervention functions and behaviour change techniques that are used within the decision-making process, as well as how these may increase participation in SDM.

A further strength of this study is the use of two researchers to extract the data from the papers and to conduct the quality assessments. This mitigates the risk of systematic bias at data extraction stage whilst also decreasing the total number of errors in data extraction and quality assessment [69]. With respect to the data extraction, online training was completed by both researchers to ensure consistency in identifying and recording behaviour change techniques. High levels of agreement were obtained, indicating strong inter-rater reliability.

A limitation of this review is that not all records were double screened, thus, whilst a high interrater reliability was reached, some articles may have been missed. A further limitation of this review is that studies did not report on the fidelity to the model or approach they were implementing. Thus, we cannot say the degree to which behaviour change techniques outlined in the papers were actually followed. As implementation has been found to affect outcomes [70], future studies into SDM interventions should report fidelity to the approach/model. In addition, most studies gave little or no information about the control group, which meant that the behaviour change techniques used here were often left unexamined. These studies also did not examine the skill of the clinician, or the amount of time they had been in the profession, which may also affect findings. Limitations of the EPHPP tool [31] also exist. Whilst it allows for comparison between randomised and non-randomised studies, some areas of bias, such as performance, assessment, and publication bias are not included. This could change the quality assessment ratings of studies if they were able to be taken into account. Finally, whilst information is provided on whether the interventions produced a statistically significant change in SDM, this does not explain if the interventions were clinically effective. Missing information in some manuscripts precluded the author’s ability to explore relationships between effect size and intervention characteristics. Researchers should consider providing information regarding effect sizes in published manuscripts in the future.

### Conclusion

To increase participation in decision making, intervention developers may wish to consider drawing on specific intervention functions and behaviour change techniques when working with stakeholders involved in the decision-making process. However, as most of the studies included in this review scored low on the EPHPP quality assessment, there is only tentative support for which behaviour change techniques and intervention function may increase participation in decision making when it comes to child and youth mental health. Future research may wish to examine findings outlined here, using more robust methods, including blinding where possible and purposefully selecting samples.

## Supplementary Information

Below is the link to the electronic supplementary material.

Supplementary file1 (DOCX 18 kb)

Supplementary file2 (DOCX 12 kb)

Supplementary file3 (DOC 68 kb)

Supplementary file4 (DOC 47 kb)

Supplementary file5 (DOC 35 kb)

Supplementary file6 (DOCX 21 kb)

Supplementary file7 (DOCX 12 kb)

## Footnotes

1 Statistical significance set at p < 0.05.

## References

[1] Ahmad N, Ellins J, Krelle H, Lawrie M (2014). Person-centred care: from ideas to action.

[2] Coulter A, Collins A (2011). Making shared decision making a reality. No decision about me, without me.

[3] Coulter A (2009). Implementing shared decision making in the UK.

[4] Charles C, Gafni A, Whelan T (1997). Shared decision-making in the medical encounter: what does it mean? (Or it takes, at least two to tango). Soc Sci Med.

[5] Alderson P (1992). In the genes or in the stars? Children’s competence to consent. J Med Ethics.

[6] United Nations (1989). Convention on the Rights of the Child.

[7] Alderson P, Montgomery J (1996). Health care choices: making decisions with children.

[8] Entwistle VA, Watt IS (2006). Patient involvement in treatment decision-making: the case for a broader conceptual framework. Patient Educ Couns.

[9] Elwyn G, Frosch D, Thomson R, Joseph-Williams N, Lloyd A, Kinnersley P (2012). Shared decision making: a model for clinical practice. J Gen Intern Med.

[10] Makoul G, Clayman M (2006). An integrative model of shared decision making in medical encounters. Patient Educ Couns.

[11] Liverpool S, Hayes D, Edbrooke-Childs J (2021). An affective-appraisal approach for parental shared decision making in children and young people’s mental health settings: a qualitative study. Front Psychiatry.

[12] Common Room Consulting. Open Talk 2017. http://www.opentalk.info/. Accessed 28 Dec 2017

[13] Wolpert M, Hoffman J, Abrines N, Feltham A, Baird L, Law D (2014). Closing the gap: Shared decision making in CAMHs. Final report for closing the gap through changing relationship.

[14] Langer D, Mooney T, Wills C (2015). Shared decision-making for treatment planning in mental health care: theory, evidence, and tools.

[15] Crickard EL, O’Brien MS, Rapp CA, Holmes CL (2010). Developing a framework to support shared decision making for youth mental health medication treatment. Community Ment Health J.

[16] Hayes D (2018). Developing an intervention to promote shared decision making in child and youth mental health: integrating theory, research and practice.

[17] Cheng H, Hayes D, Edbrooke-Childs J, Martin K, Chapman L, Wolpert M (2017). What approaches for promoting shared decision making are used in child mental health? A scoping review. Clin Psychol Psychother.

[18] Liverpool S, Pereira B, Hayes D, Wolpert M, Edbrooke-Childs J (2020). A scoping review and assessment of essential elements of shared decision-making of parent-involved interventions in child and adolescent mental health. Eur Child Adolesc Psychiatry.

[19] Davis R, Campbell R, Hildon Z, Hobbs L, Michie S (2015). Theories of behaviour and behaviour change across the social and behavioural sciences: a scoping review. Health Psychol Rev.

[20] Albada A, Ausems MG, Bensing JM, van Dulmen S (2009). Tailored information about cancer risk and screening: a systematic review. Patient Educ Couns.

[21] Noar SM, Benac CN, Harris MS (2007). Does tailoring matter? Meta-analytic review of tailored print health behavior change interventions. Psychol Bull.

[22] Sheehan J, Sherman KA (2012). Computerised decision aids: a systematic review of their effectiveness in facilitating high-quality decision-making in various health-related contexts. Patient Educ Couns.

[23] Charles C, Gafni A, Whelan T (1999). Decision-making in the physician–patient encounter: revisiting the shared treatment decision-making model. Soc Sci Med.

[24] Elwyn G, Edwards A, Gwyn R, Grol R (1999). Towards a feasible model for shared decision making: focus group study with general practice registrars. Br Med J.

[25] Legare F, Thompson-Leduc P (2014). Twelve myths about shared decision making. Patient Educ Couns.

[26] Towle A, Godolphin W (1999). Framework for teaching and learning informed shared decision making. Br Med J.

[27] Michie S, Atkins L, West W (2014). The behaviour change wheel: a guide to designing interventions.

[28] Légaré F, Stacey D, Turcotte S, Cossi MJ, Kryworuchko J, Graham ID (2014). Interventions for improving the adoption of shared decision making by healthcare professionals. Cochrane Database Syst Rev.

[29] Agbadjé TT, Elidor H, Perin MS, Adekpedjou R, Légaré F (2020). Towards a taxonomy of behavior change techniques for promoting shared decision making. Implement Sci.

[30] Ruhe KM, Wangmo T, Badarau DO, Elger BS, Niggli F (2015). Decision-making capacity of children and adolescents—suggestions for advancing the concept’s implementation in pediatric healthcare. Eur J Pediatr.

[31] Thomas BH, Ciliska D, Dobbins M, Micucci S (2004). A process for systematically reviewing the literature: providing the research evidence for public health nursing interventions. Worldviews Evid Based Nurs.

[32] Deeks JJ, Dinnes J, D’amico R, Sowden AJ, Sakarovitch C, Song F (2003). Evaluating non-randomised intervention studies. Health Technol Assess (Rockv).

[33] Aoki Y, Takaesu Y, Inoue M, Furuno T, Kobayashi Y, Chiba H (2019). Seven-day shared decision making for outpatients with first episode of mood disorders among university students: a randomized controlled trial. Psychiatry Res.

[34] Simmons M, Batchelor S, Dimopoulos-Bick T, Howe D (2017). The choice project: peer workers promoting shared decision making at a youth mental health service. Psychiatr Serv.

[35] Rowe SL, Patel K, French RS, Henderson C, Ougrin D, Slade M (2018). Web-based decision aid to assist help-seeking choices for young people who self-harm: outcomes from a randomized controlled feasibility trial. JMIR Ment Heal.

[36] Walker JS, Seibel CL, Jackson S (2017). Increasing youths’ participation in team-based treatment planning: the achieve my plan enhancement for wraparound. J Child Fam Stud.

[37] Brinkman WB (2011). Physicians’ shared decision-making behaviors in attention-deficit/hyperactivity disorder care. Arch Pediatr Adolesc Med.

[38] Westermann GMA, Verheij F, Winkens B, Verhulst FC, Van Oort FVA (2013). Structured shared decision-making using dialogue and visualization: a randomized controlled trial. Patient Educ Couns.

[39] Grant N (2016). Assisting parents of children with autism to make intervention decisions by improving their health literacy about evidence.

[40] Hogue A, Lichvar E, Bobek M (2016). Evaluation of the medication integration protocol for adolescents with ADHD in behavioral care: treatment fidelity and medication uptake. J Emot Behav Disord.

[41] Brinkman WB, Hartl Majcher J, Poling LM, Shi G, Zender M, Sucharew H (2013). Shared decision-making to improve attention-deficit hyperactivity disorder care. Patient Educ Couns.

[42] O’Connor A (2006) Ottawa decision support framework to address decisional conflict. Ottawa 10.1177/0272989X06290492 16855126

[43] Bernoulli D (1954). Exposition of a new theory on the measurement of risk. (Original 1738). Econometrica.

[44] Howard RA, Matheson JE (1984). Readings on the principles and applications of decision analysis.

[45] Kahneman D, Tversky A (1979). Prospect theory: an analysis of decision under risk. Econometrica.

[46] Janis IL, Mann L, Greenwald A, Brock T, Ostrom T (1968). A conflict-theory approach to attitude change and decision making. Psychol. Found attitudes.

[47] Fishbein M, Ajzen I (1975) Belief, attitude, intention and behavior: an introduction to theory and research. Addison-Wesley, Reading

[48] Bandura A (1986). Social foundations of thought and action: a social cognitive theory.

[49] Norbek JS (1988). Social support. Nurs Res.

[50] Orem DE (1995). Nursing: concepts of practice.

[51] O’Connor AM, Llewellyn-Thomas H, Stacey D (2005) IPDAS Collaboration Background Document. Available from www.ipdas.ohri.ca. Accessed 2 Apr 2018

[52] Brinkman WB, Sherman SN, Zmitrovich AR, Visscher MO, Crosby LE, Phelan KJ (2012). In their own words: adolescent views on ADHD and their evolving role managing medication. Acad Pediatr.

[53] McDonald H, Charles C, Gafni A (2014). Assessing the conceptual clarity and evidence base of quality criteria/standards developed for evaluating decision aids. Heal Expect.

[54] Bekker HL (2010). The loss of reason in patient decision aid research: do checklists damage the quality of informed choice interventions?. Patient Educ Couns.

[55] Durand MA, Stiel M, Boivin J, Elwyn G (2008). Where is the theory? Evaluating the theoretical frameworks described in decision support technologies. Patient Educ Couns.

[56] Bowen DJ, Allen JD, Vu T, Johnson RE, Fryer-Edwards K, Hart A (2006). Theoretical foundations for interventions designed to promote informed decision making for cancer screening. Ann Behav Med.

[57] Elwyn G, Stiel M, Durand MA, Boivin J (2011). The design of patient decision support interventions: Addressing the theory-practice gap. J Eval Clin Pract.

[58] Cane J, O’Connor D, Michie S (2012). Validation of the theoretical domains framework for use in behaviour change and implementation research. Implement Sci.

[59] Hayes D, Edbrooke-Childs J, Town R, Wolpert M, Midgley N (2019). Barriers and facilitators to shared decision-making in child and youth mental health: exploring young person and parent perspectives using the theoretical domains framework. Couns Psychother Res.

[60] Gondek D, Edbrooke-Childs J, Velikonja T, Chapman L, Saunders F, Hayes D (2016). Facilitators and barriers to person-centred care in child and young people mental health services: a systematic review. Clin Psychol Psychother.

[61] Slade M (2017). Implementing shared decision making in routine mental health care. World Psychiatry.

[62] Simmons M, Hetrick S, Jorm AF (2011). Experiences of treatment decision making for young people diagnosed with depressive disorders: a qualitative study in primary care and specialist mental health settings. BMC Psychiatry.

[63] Hayes D, Edbrooke-Childs J, Town R, Wolpert M, Midgley N (2018). Barriers and facilitators to shared decision making in child and youth mental health: clinician perspectives using the theoretical domains framework. Eur Child Adolesc Psychiatry.

[64] Meader N, King K, Wright K, Graham HM, Petticrew M, Power C (2017). Multiple risk behavior interventions: meta-analyses of RCTs. Am J Prev Med.

[65] Stacey D, Légaré F, Lewis K, Barry MJ, Bennett CL, Eden KB (2017). Decision aids for people facing health treatment or screening decisions (review). Cochrane Database Syst Rev.

[66] Abrines-Jaume N, Midgley N, Hopkins K, Hoffman J, Martin K, Law D (2016). A qualitative analysis of implementing shared decision making in child and adolescent mental health services in the United Kingdom: stages and facilitators. Clin Child Psychol Psychiatry.

[67] Duncan E, Best C, Hagen S (2010). Shared decision making interventions for people with mental health conditions. Cochrane Database Syst Rev.

[68] Butler AM, Weller B, Titus C (2015). Relationships of shared decision making with parental perceptions of child mental health functioning and care. Adm Policy Ment Heal Ment Heal Serv Res.

[69] Buscemi N, Hartling L, Vandermeer B, Tjosvold L, Klassen TP (2006). Single data extraction generated more errors than double data extraction in systematic reviews. J Clin Epidemiol.

[70] Durlak JA, DuPre EP (2008). Implementation matters: a review of research on the influence of implementation on program outcomes and the factors affecting implementation. Am J Community Psychol.

