# Supplementary material for: A systematic review of shared decision making interventions in child and youth mental health: synthesising the use of theory, intervention functions, and behaviour change techniques
Source: Eur Child Adolesc Psychiatry. 2021 Apr 22;32(2):209–22. doi: 10.1007/s00787-021-01782-x (PMC9970944; doi:10.1007/s00787-021-01782-x)
Supplement: Supplementary file 1 — Supplementary file1 (DOCX 18 kb) [file 787_2021_1782_MOESM1_ESM.docx]

| **Included article number** | **Author, year of publication, and country** | **N** | **Comparison and design** | **Process measure: decision making/ involvement/ participation** |
| --- | --- | --- | --- | --- |
| 1. | Aoki et al., (2020), Japan. | 88 young people with a mood (depression or bipolar) disorder  35 intervention and 53 usual care | Intervention vs usual practice  Randomised control trial | Significant increase in SDM (COMRADE; Edwards et al., 2003: YP rated)  Intervention: 44(9)*  Control: 38 (7)*  p< 0.001 |
| 2. | Brinkman (2013),  US | 44 parents/guardians of young people with ADHD, 7 paediatricians  21 control and 33 intervention | Intervention vs usual practice  Controlled clinical trial | Increase in SDM (Option Scale; Elwyn et al.,  2005: O rated)  Intervention: 43.8 (5.6)  Control: 31.2 (3.7)  p<0.001 |
| 3. | Grant (2016), Australia | 81 parents/guardians of young people with autism  42 control and 29 intervention | Intervention vs usual practice | No significant increase in decisional conflict^#^ (Support subscale: P/G rated)  Intervention: 34.5 (22.4)  Control: 36.4 (24.6)  p=.42 |
| 4. | Hogue et al. (2016),  US | 3 MIP therapists and 119 young people with ADHD and their parents/guardians  39 control and 80 intervention | Intervention vs historical control  Controlled clinical trial | Significant increase in family decision making (O rated)  Non validated measure  Intervention 1.8 (1.1)  Control 1 (0.00)  p< 0.00 |
| 5. | Rowe et al., (2018) UK | 23 young people with self harm  13 in control and 10 in intervention | Intervention vs usual practice.  Randomised Control Trial | No difference in decisional conflict ^#^ (Support subscale: YP rated)  Intervention 21.7 (26.1)  Control 15.4 (18.6)  p=.55 |
| 6. | Simmons et al., (2017) Australia | 149 young people ages 16-25 (presenting difficulty not specific),  61 in control and 78 in intervention | Intervention vs historical control  Controlled clinical trial | Significant increase in SDM (SDM-Q-9-[43] (YP rated)  Intervention 38.81 (7.41)  Control 37.97 (5.28)  p<0.015 |
| 7. | Walker et al., (2017). US | 55 high risk young people with mental health difficulties (not specific). Involved in at least two systems designed to support young people (mental health and child welfare)  20 control and 35 intervention | Intervention vs usual practice.  Randomised Control Trial | Increase Participation  Youth Participation in Planning Scale (YPP; Walker and Powers 2007) (YP rated)  Intervention: not available  Control: not available  p<0.00 |
| 8. | Westermann et al., (2013). Netherlands | parents/guardians of young people (presenting difficulty not specific), and 20 therapists  61 in control and 72 in intervention | SDM vs usual practice:  Randomised Control Trials | Increase in satisfaction with participation in shared decision making (mothers)  Post scores  p<0.03 |

^*^ Median and IQR. ^#^ The decisional conflict scale (O’Connor, 1995) is both a process and outcome measure for SDM [44]. YP rated – young person rated. P/G rated – parent/guardian rated. O rated – observer rated. T rated – teacher rated. C rated – Clinician rated.
