# Supplementary material for: A systematic review of shared decision making interventions in child and youth mental health: synthesising the use of theory, intervention functions, and behaviour change techniques
Source: Eur Child Adolesc Psychiatry. 2021 Apr 22;32(2):209–22. doi: 10.1007/s00787-021-01782-x (PMC9970944; doi:10.1007/s00787-021-01782-x)
Supplement: Supplementary file 2 — Supplementary file2 (DOCX 12 kb) [file 787_2021_1782_MOESM2_ESM.docx]

**Search strategy**

| **Search strategy** |
| --- |
| **SDM**  (("Shared decision making" OR "Self determination " OR "Client choice " OR "Informed choice " OR "Patient choice" OR "Client participation" OR Decision-aids OR ("Client cent* care" OR "patient cent* care" ) OR "Therapeutic alliance " OR "Collaborative practice" OR "Recovery oriented care " OR "Shared care" OR "User empowerment" OR "Shared agreements " OR "Common goals " OR "Value oriented care " OR Personali*ation)  *AND*  **Child or young person**  (Child* OR "young person* " OR teen* OR adolescen* OR tween* OR "high school" OR "secondary school" OR "primary school" OR juvenile OR "Elementary school" OR Student* OR "Middle school" OR "Nursery school" OR Pre-school )  *AND*  **Mental health**  ("exp Mental health" OR "exp Mental illness" OR "exp Mental disorder*" OR "Chronic mental illness" OR "Community mental health" OR "Community mental health cent*" OR "Community mental health service*" OR "Primary mental health prevention" OR "Anxiety disorder* " OR "Anxiety management" OR "Emotional problem* " OR "Emotional adjustment" OR "Affective disorder* " OR "Behavio*r disorders" OR "Behavio*r problem* " OR "child psychopathology" OR Psychosis OR (Neurosis or neuroses) OR "Cognitive behavio*r therapy" OR depressi* OR Psycholog* OR Therapy OR Counselling))*]* |
