# Supplementary material for: A systematic review of shared decision making interventions in child and youth mental health: synthesising the use of theory, intervention functions, and behaviour change techniques
Source: Eur Child Adolesc Psychiatry. 2021 Apr 22;32(2):209–22. doi: 10.1007/s00787-021-01782-x (PMC9970944; doi:10.1007/s00787-021-01782-x)
Supplement: Supplementary file 3 — Supplementary file3 (DOC 68 kb) [file 787_2021_1782_MOESM3_ESM.doc]

Table 1 Behaviour change techniques present in studies and whether the study found increased SDM

|  |  |  |  |  |  |  |  |  |  |  |  |  |  |  |  |  |  |  |
| --- | --- | --- | --- | --- | --- | --- | --- | --- | --- | --- | --- | --- | --- | --- | --- | --- | --- | --- |
| **Study and intervention** | **Target group** | **1.2** | **1.3** | **1.5** | **1.7** | **3.1** | **3.2** | **4.1** | **5.1** | **5.3** | **6.1** | **7.1** | **8.1** | **8.3** | **9.1** | **9.2** | **12.5** | **13.2** |
| **DECISION AIDS** | | | | | | | | | | | | | | | | | | |
| **Aoki et al. (2020)** | Young people |  |  |  |  |  |  |  | ✓ |  |  |  |  |  | ✓ | ✓ | ✓ |  |
|  | Clinicians |  |  |  |  |  |  | ✓ |  |  |  |  | ✓ | ✓ |  |  | ✓ |  |
| **Brinkman et al. (2013)** | Parents/  guardians |  | ✓ |  |  |  |  | ✓ | ✓ |  |  |  |  |  | ✓ | ✓ | ✓ |  |
|  | Clinician |  |  |  |  |  |  |  |  |  | ✓ | ✓ |  |  |  |  | ✓ |  |
| **Grant (2016)** | Parents/  guardians |  |  |  |  |  |  |  |  |  |  |  |  |  |  | X | X |  |
| **Simmons et al. (2017).** | Young people |  |  |  |  | ✓ |  |  |  |  |  |  |  |  | ✓ | ✓ | ✓ |  |
|  | Peer worker |  |  |  |  |  |  | ✓ |  |  |  |  | ✓ | ✓ | ✓ |  | ✓ |  |
| **Rowe et al. (2018)** | Young people |  |  |  |  |  |  |  |  |  |  |  |  |  |  | X | X |  |
| **THERAPEUTIC APPROACHES** | | | | | | | | | | | | | | | | | | |
| **Hogue et al. (2016)** | Young people | ✓ | ✓ | ✓ | ✓ |  |  |  | ✓ | ✓ |  |  |  |  | ✓ | ✓ |  | ✓ |
|  | Parents/ guardians | ✓ | ✓ | ✓ | ✓ |  |  |  | ✓ | ✓ |  |  |  |  | ✓ | ✓ |  | ✓ |
|  | Clinician |  |  |  |  |  |  |  |  |  |  |  | ✓ | ✓ |  |  |  |  |
| **Walker et al. (2017** | Young people | ✓ | ✓ |  | ✓ |  | ✓ |  |  |  |  |  |  |  |  |  |  |  |
|  | Decision coach |  |  |  |  |  |  | ✓ |  |  |  |  | ✓ | ✓ |  |  |  |  |
| **Westermann et al. (2013).** | Parents/ guardians | ✓ |  |  |  |  |  |  |  |  |  |  |  |  | ✓ | ✓ | ✓ |  |
|  | Clinician | ✓ |  |  |  |  |  |  |  |  |  |  | ✓ | ✓ |  | ✓ | ✓ |  |

1.2 Problem solving, 1.3 Goal setting (outcome), 1.5 Review (behavioural) goals, 1.7 Review (outcome) goals, 3.1 Social support (unspecified), 3.2 Social support (practical), 4.1 Instructions on how to perform the behaviour, 5.1 Information about health consequences, 5.3 Information about social and environmental consequences, 6.1 Demonstration of the behaviour, 7.1 Prompts/cues, 8.1 Behavioural practice/rehearsal, 8.3 Habit formation, 9.1 Credible source, 9.2 Pros and cons, 12.5 Adding objects to the environment, 13.2 Framing/reframing, ✓ Study reported an increase in decision making participation, X Study reported no increase in decision making participation
