# Supplementary material for: A systematic review of shared decision making interventions in child and youth mental health: synthesising the use of theory, intervention functions, and behaviour change techniques
Source: Eur Child Adolesc Psychiatry. 2021 Apr 22;32(2):209–22. doi: 10.1007/s00787-021-01782-x (PMC9970944; doi:10.1007/s00787-021-01782-x)
Supplement: Supplementary file 4 — Supplementary file4 (DOC 47 kb) [file 787_2021_1782_MOESM4_ESM.doc]

Table 2: Intervention functions present and whether the study findings indicated an increase in SDM

|  | | **Intervention functions** | | | | |
| --- | --- | --- | --- | --- | --- | --- |
| **Study and intervention** | **Target group** | **Education** | **Training** | **Modelling** | **Environmental Restructuring** | **Enablement** |
| **DECISION AIDS** | | | | | | |
| **Aoki et al. (2020)** | Young people | ✓ |  |  | ✓ | ✓ |
|  | Clinicians | ✓ | ✓ |  | ✓ |  |
| **Brinkman et al. (2013)** | Parents/guardians | ✓ |  |  | ✓ | ✓ |
|  | Clinician | ✓ | ✓ | ✓ | ✓ | ✓ |
| **Grant (2016)** | Parents/guardians | X |  |  | X | X |
| **Rowe et al. (2018).** | Young people | X |  |  | X | X |
| **Simmons et al. (2017). Decision aid and peer support** | Young people | ✓ |  |  | ✓ | ✓ |
|  | Peer workers | ✓ | ✓ |  | ✓ | ✓ |
| **THERAPEUTIC APPROACHES** | | | | | | |
| **Hogue et al. (2016)** | Young people | ✓ |  |  |  | ✓ |
|  | Parents/guardians | ✓ |  |  |  | ✓ |
|  | Clinician | ✓ | ✓ |  |  |  |
| **Walker et al. (2017).** | Young people |  |  |  |  | ✓ |
|  | Health coach | ✓ | ✓ |  |  |  |
| **Westermann et al. (2013). Counselling in Dialogue.** | Parents/guardians | ✓ |  |  | ✓ | ✓ |
|  | Clinician | ✓ | ✓ | ✓ | ✓ | ✓ |

✓ Study reported an increase in decision making participation, X Study reported no increase in decision making participation
