# Supplementary material for: A systematic review of shared decision making interventions in child and youth mental health: synthesising the use of theory, intervention functions, and behaviour change techniques
Source: Eur Child Adolesc Psychiatry. 2021 Apr 22;32(2):209–22. doi: 10.1007/s00787-021-01782-x (PMC9970944; doi:10.1007/s00787-021-01782-x)
Supplement: Supplementary file 5 — Supplementary file5 (DOC 35 kb) [file 787_2021_1782_MOESM5_ESM.doc]

| Paper | **Selection Bias** | **Study design** | **Confounders** | **Blinding** | **Data Collection** | **Withdrawal and Dropout** | **Overall** |
| --- | --- | --- | --- | --- | --- | --- | --- |
| Aoki et al. (2020) | Weak | Strong | Weak | Moderate | Strong | N/A | Weak |
| Brinkman et al. (2013) | Moderate | Strong | Weak | Weak | Strong | Strong | Weak |
| Grant (2016) | Weak | Strong | Strong | Moderate | Strong | Strong | Moderate |
| Hogue et al.  (2016) | Moderate | Moderate | Weak | Weak | Moderate | N/A | Weak |
| Rowe et al., (2018) | Weak | Strong | Weak | Moderate | Strong | Strong | Weak |
| Simmons et al., (2017) | Moderate | Moderate | Strong | Weak | Moderate | N/A | Moderate |
| Walker et al., (2017) | Weak | Strong | Strong | Weak | Strong | Moderate | Weak |
| Westermann et al., (2013) | Strong | Strong | Strong | Moderate | Strong | Strong | Strong |

Table 3: EPHPP Quality Assessment Method for risk of bias[28]
