# Supplementary material for: A systematic review of shared decision making interventions in child and youth mental health: synthesising the use of theory, intervention functions, and behaviour change techniques
Source: Eur Child Adolesc Psychiatry. 2021 Apr 22;32(2):209–22. doi: 10.1007/s00787-021-01782-x (PMC9970944; doi:10.1007/s00787-021-01782-x)
Supplement: Supplementary file 6 — Supplementary file6 (DOCX 21 kb) [file 787_2021_1782_MOESM6_ESM.docx]

| BCT | Description | Example |
| --- | --- | --- |
| 1.2 Problem solving | Analyse, or prompt the person to analyse, factors influencing the behaviour and generate or select strategies that include overcoming barriers and/or increasing facilitators | Therapist works with young person and family to explore and understand environmental factors  that support or impede school and treatment success (Hogue et al., 2016) |
| 1.3 Goal setting (outcome) | Set or agree on a goal defined in terms of a positive outcome of wanted behaviour | Patients are given a card and asked down to write their goals for treatment (Brinkman et al., 2013) |
| 1.5 Review (behavioural) goals | Review behaviour goal(s) jointly with the person and consider modifying goal(s) or behaviour change strategy in light of achievement. | Therapists revisit goal with family around medication management and taking (Hogue et al., 2013) |
| 1.7 Review (outcome) goals | Review outcome goal(s) jointly with the person and consider modifying goal(s) in light of achievement | Therapists revisit goal with family around medication initiation and management (Hogue et al., 2016) |
| 3.1 Social support (unspecified) | Advise on, arrange or provide social support (e.g., from friends, relatives, colleagues,’ buddies’ or staff) or noncontingent praise or reward for performance of the behaviour | Peer support workers greet client, provide assistance before and after sessions (Simmons et al., 2017). |
| 3.2 Social support (practical) | Advise on, arrange, or provide practical help (e.g., from friends, relatives, colleagues, ‘buddies’ or staff) for performance of the behaviour | Team members are asked by young people for support during wrap around meetings to help achieve goals s needed (Walker et al., 2017). |
| 4.1 Instructions on how to perform the behaviour | Advise or agree on how to perform the behaviour | Clinicians was provided with a one-day training and instructions on how to use decision aid (Aoki, et al., 2020) |
| 5.1 Information about health consequences | Provide information (e.g., written, verbal, visual) about health consequences of performing the behaviour | Decision aids, provided by clinicians, went through options associated with treatment and outcomes (e.g. chances of side effects) (Brinkman et al., 2013) |
| 5.3 Information about social and environmental consequences | Provide information (e.g., written, verbal, visual) about health consequences of performing the behaviour | Therapist helps family understand the potential benefits of  Medication n home, school, and peer contexts (Hogue et al., 2016) |
| 6.1 Demonstration of the behaviour | Provide an observable sample of the performance of the behaviour, directly in person or indirectly e.g., via film, pictures, for the person to aspire to or imitate | Clinicians are provided with a demonstration of how decision aid should be used (Brinkman et al., 2013). |
| 7.1 Prompts/cues | Introduce or define environmental or social stimulus with the purpose of prompting or cueing the behaviour. The prompt or cue would normally occur at the time or place of performance | Prior to entering the examination room for each study encounter, physicians were handed the parent-completed goal/preference card,  behavioral treatment card, and the ADHD medication choice cards. Physicians were  reminded to ask parents to pick a medication choice card (Brinkman et al., 2013). |
| 8.1 Behavioural practice/rehearsal | Prompt practice or rehearsal of the performance of the behaviour one or more times in a context or at a time when the performance may not be necessary, in order to increase habit and skill | As part of training, clinicians underwent role-plays (Aoki et al., 2020) |
| 8.3 Habit formation | Prompt rehearsal and repetition of the behaviour in the same context repeatedly so that the context elicits the behaviour | In addition to role plays, booster sessions were provided (Aoki et al., 2020) |
| 9.1 Credible source | Present verbal or visual communication from a credible source in favour of or against the behaviour | Clinicians, by view of their credentials/professional role, were seen as experts in treatment options |
| 9.2 Pros and cons | Advise the person to identify and compare reasons for wanting (pros) and not wanting to (cons) change the behaviour | Treatment options were presented side-by-side and pros/cons were explored of each (Brinkman et al., 2013) |
| 12.5 Adding objects to the environment | Add objects to the environment in order to facilitate performance of the behaviour | Web-based decision aids were provided to help young people make decisions (Rowe et al., 2013). |
| 13.2 Framing/reframing | Suggest the deliberate adoption of a perspective or new perspective on behaviour (e.g., its purpose) in order to change cognitions or emotions about performing the behaviour | Therapists provided different lenses for young people and family to redefine and reframe problems and solutions (Hogue et al., 2016). |
