# Supplementary material for: A systematic review of shared decision making interventions in child and youth mental health: synthesising the use of theory, intervention functions, and behaviour change techniques
Source: Eur Child Adolesc Psychiatry. 2021 Apr 22;32(2):209–22. doi: 10.1007/s00787-021-01782-x (PMC9970944; doi:10.1007/s00787-021-01782-x)
Supplement: Supplementary file 7 — Supplementary file7 (DOCX 12 kb) [file 787_2021_1782_MOESM7_ESM.docx]

| Intervention function | Description | Example |
| --- | --- | --- |
| Education | Increasing knowledge or understanding | The therapist provides psychoeducation around ADHD to the family (Hogue et al., 2016) |
| Training | Imparting skills | Clinicians are taught the skills needed to enact the therapeutic technique (Westermann 2013) |
| Environmental restructuring | Changing the physical or social context | Providing a decision aid during a clinical appointment, so young people could make an informed decision (Simmons et al., 2017) |
| Modelling | Providing an example for people to aspire to or imitate | Therapists see how to enact the therapeutic technique (Westermann 2013) |
| Enablement | Increasing means/reducing barriers to increase capability or opportunity | Therapist reframing questions so that different perspectives can be seen by family members (Hogue et al., 2016) |

Table 5: A description of intervention functions and examples within included studies
